# Mechanism and Physiologic Significance of the Suppression of Cholesterol Esterification in Human Interstitial Fluid

**DOI:** 10.3389/fphar.2016.00216

**Published:** 2016-07-15

**Authors:** Norman E. Miller, Waldemar L. Olszewski, Irina P. Miller, Mahmud N. Nanjee

**Affiliations:** ^1^Magdalen College, University of Oxford, OxfordUK; ^2^Department of Surgical Research and Transplantology, Medical Research Centre, Polish Academy of Sciences, WarsawPoland; ^3^Department of Cardiovascular Biochemistry, Queen Mary University of London, LondonUK; ^4^Cardiovascular Genetics Unit, School of Medicine, University of Utah, Salt Lake City, UTUSA

**Keywords:** apolipoprotein AI, lecithin:cholesterol acyltransferase, high density lipoproteins, lymph, interstitial fluid

## Abstract

Cholesterol esterification in high density lipoproteins (HDLs) by lecithin:cholesterol acyltransferase (LCAT) promotes unesterified cholesterol (UC) transfer from red cell membranes to plasma *in vitro*. However, it does not explain the transfer of UC from most peripheral cells to interstitial fluid *in vivo*, as HDLs in afferent peripheral lymph are enriched in UC. Having already reported that the endogenous cholesterol esterification rate (ECER) in lymph is only 5% of that in plasma, we have now explored the underlying mechanism. In peripheral lymph from 20 healthy men, LCAT concentration, LCAT activity (assayed using an optimized substrate), and LCAT specific activity averaged, respectively, 11.8, 10.3, and 84.9% of plasma values. When recombinant human LCAT was added to lymph, the increments in enzyme activity were similar to those when LCAT was added to plasma. Addition of apolipoprotein AI (apo AI), fatty acid-free albumin, Intralipid, or the *d* < 1.006 g/ml plasma fraction had no effect on ECER. During incubation of lymph plus plasma, the ECER was similar to that observed with buffer plus plasma. When lymph was added to heat-inactivated plasma, the ECER was 11-fold greater than with lymph plus buffer. Addition of discoidal proteoliposomes of apo AI and phosphatidycholine (PC) to lymph increased ECER 10-fold, while addition of apo AI/PC/UC disks did so by only six-fold. We conclude that the low ECER in lymph is due to a property of the HDLs, seemingly substrate inhibition of LCAT by excess cell-derived UC. This is reversed when lymph enters plasma, consequent upon redistribution of UC from lymph HDLs to plasma lipoproteins.

## Introduction

Glomset et al. (1966) showed that the unesterified cholesterol (UC) of high density lipoproteins (HDLs) is the optimum physiologic substrate for lecithin:cholesterol acyltransferase (LCAT) in plasma. He also showed that cholesterol esterification in HDLs promotes the transfer of UC from red cell membranes to plasma HDLs *in vitro* (Glomset, 1970; Glomset and Norum, 1973). On this basis, he hypothesized that the HDL-LCAT system provides the mechanism by which UC is drawn from peripheral cells to HDLs for delivery to the liver and subsequent elimination. However, later observations seemed to conflict with this concept. In humans and mice with genetic LCAT deficiencies, for example, excess cholesterol was found to be restricted to specific sites, including red blood cells, renal glomeruli, and the spleen (Stokke et al., 1974; Lambert et al., 2001). Based on studies in transgenic mice, Alam et al. (2001) and Tanigawa et al. (2009) concluded that variations in LCAT gene expression had little or no effect on reverse cholesterol transport. Reports that the rate of esterification of UC is much lower in peripheral lymph than in plasma (Dory et al., 1983; Miller et al., 2013) also questioned the role of LCAT in cholesterol removal from most cells. Confirmation that LCAT cannot be rate-limiting was provided by demonstrations that the UC/CE ratio in lymph HDLs exceeds that in plasma HDLs (Reichl et al., 1980; Nanjee et al., 2000b), and that the former include disks devoid of core lipids (Nanjee et al., 2001) similar to those seen in the plasma of LCAT-deficient subjects (Glomset et al., 1980). While this line of research was progressing, others discovered the role of ABCA1 transporters in mediating the egress of UC from cells to lipid-poor apo AI particles (Oram and Heinecke, 2005).

The explanation of the profound suppression of cholesterol esterification in HDLs once they have transferred from plasma across endothelium into the interstitial fluid is not known. Apolipoprotein (apo) AI, the principal cofactor of LCAT (Fielding et al., 1972), has been shown to be present at a concentration of about one fifth that in plasma (Nanjee et al., 2000b), but there are no published data on LCAT concentration in lymph. The UC in discoidal HDLs isolated from the plasma of subjects with familial LCAT deficiency is an efficient substrate for the enzyme (Glomset et al., 1970). This is also true of UC acquired by reconstituted HDL disks composed of phosphatidylcholine (PC) and human apo AI (Nanjee et al., 1999), and of the UC in HDL disks recovered from rat liver perfusates (Hamilton et al., 1976). When infused intravenously into humans, apo AI/PC disks acquire UC and are rapidly converted to CE-rich spheroidal HDLs (Nanjee et al., 1999). And yet the discoidal HDLs formed in interstitial fluid do not undergo such metabolism until after they have entered the blood.

An understanding of the mechanism of the suppression of cholesterol esterification in interstitial fluid may shed light on the regulation of LCAT, and aid the development of new approaches to the prevention of atherosclerosis by modulation of HDL metabolism. In this the first study of the mechanism in humans, we have tested several hypotheses by experiments *in vitro* with normal human afferent lymph, which is representative of interstitial fluid (Nanjee et al., 2000b, 2001).

## Materials and Methods

### Subjects

Lymph from 20 healthy men was studied. Ages were 20–69 years (mean, 34.5 years), body weights 60–97 kg (75 kg), body mass indexes 19.3–28.4 kg/m^2^ (23.5 kg/m^2^), plasma total cholesterol 1.54–5.48 mmol/l (4.16 mmol/l), plasma HDL cholesterol 0.71–1.89 mmol/l (1.22 mmol/l), and plasma triglycerides 0.74–3.26 mmol/l (1.36 mmol/l). Screening of the volunteers for health status was as previously described (Nanjee et al., 2000b). All subjects gave informed consent. The protocol had been approved by the ethics committees of London Bridge Hospital and St Bartholomew’s Hospital Medical School, London, where the clinical and laboratory procedures were performed, respectively. All subjects gave written informed consent.

### Clinical Procedures

Interstitial fluid was collected as afferent (pre-nodal) peripheral lymph from overnight-fasted subjects under metabolic ward conditions by cannulation of a lymph vessel in the lower leg, according to our previously described procedures (Nanjee et al., 2000b). Lymph was collected for periods of 2 or 3 h into plastic tubes containing 2 mg dry disodium EDTA (Nanjee et al., 2000b). Flow rates were 0.10–2.0 ml/h (mean 0.78). Preliminary studies had shown that collection into tubes containing an LCAT inhibitor, or collection into tubes chilled in crushed ice, had no effect on the UC/CE ratios of the samples (Nanjee et al., 2000b; Cooke et al., 2004), indicating there was essentially no esterification of cholesterol during the collection period. Venous blood samples were collected into disodium EDTA (1 mg/ml) at the mid-point of each lymph collection period, and placed into crushed ice.

### Laboratory Procedures

Blood and lymph samples were centrifuged immediately after collection, as previously described (Nanjee et al., 2000b). The supernatants were divided into aliquots, which were flash frozen in liquid nitrogen, and then transferred to a freezer at -85°C. All assays were performed in duplicate, and the mean result calculated. The details of the laboratory procedures used in each experiment are provided as footnotes to the figures and table.

## Results

### Endogenous Cholesterol Esterification Rate

In conformity with previous studies, the endogenous cholesterol esterification rate (ECER) in lymph during incubation *in vitro* averaged only 4.2% (*P* < 0.001) of that in plasma from the same subjects (**Table 1**). The rate was not correlated with lymph LCAT concentration (*r* = +0.06), lymph LCAT activity (*r* = –0.02), or lymph apo AI concentration (*r* = +0.02).

**Table 1:** Endogenous esterification rate, LCAT activity, LCAT concentration, LCAT specific activity, and apo AI concentration in lymph and plasma samples from twenty healthy male subjects.

	LCAT conc^1^ (μg/ml)		ECER^2^ (nmol/ml/h)		LCAT activity^3^ (nmol/ml/h)		LCAT sp activity^4^ (nmol/h/μg)		Apo AI conc^5^ (mg/ml)		UC conc^6^ (mmol/I)	
Subject	Lymph	Plasma	Lymph	Plasma	Lymph	Plasma	Lymph	Plasma	Lymph	Plasma	Lymph	Plasma
1	0.84	5.24	2.13	69.6	13.5	103	16.1	19.7	0.24	1.02	0.13	1.17
2	0.71	6.46	3.70	69.3	12.8	126	18.0	19.5	0.19	0.85	0.05	1.02
3	1.06	6.20	2.01	37.6	20.4	119	19.2	19.2	0.32	1.19	0.14	1.01
4	0.54	6.26	2.62	57.0	10.4	132	19.3	21.1	0.13	0.77	0.06	1.06
5	0.58	6.05	1.27	48.2	7.40	104	12.8	17.2	0.17	0.86	0.05	0.86
6	1.08	7.08	2.26	44.7	14.9	105	13.8	14.9	0.19	0.82	0.08	0.80
7	0.76	8.62	3.52	68.5	10.1	141	13.3	16.4	0.20	1.29	0.10	1.30
8	0.96	8.55	3.62	83.3	12.0	133	12.5	15.5	0.23	0.87	0.10	1.16
9	1.32	8.19	4.60	46.1	20.9	155	15.8	18.9	0.30	0.97	0.09	0.90
10	0.68	6.34	4.19	70.6	7.30	131	10.7	20.7	0.17	0.99	0.07	0.94
11	0.37	6.86	2.88	57.3	5.00	125	13.5	18.3	0.09	0.90	0.04	1.04
12	1.32	7.42	1.77	58.4	23.8	132	18.0	17.8	0.33	1.18	0.11	0.89
13	0.84	6.99	1.54	57.0	13.5	129	16.1	18.4	0.17	0.81	0.13	1.11
14	0.74	8.78	2.28	65.2	11.2	179	15.1	20.3	0.28	1.51	0.07	1.05
15	1.66	7.91	3.16	66.4	24.2	141	14.6	17.8	0.34	0.91	0.23	1.08
16	0.61	4.95	1.38	52.3	10.3	96	16.9	19.4	0.16	0.78	0.07	0.94
17	0.71	10.9	1.25	68.6	8.90	154	12.5	14.2	0.13	0.87	0.05	1.31
18	0.79	7.96	5.10	79.3	10.9	142	13.8	17.8	0.28	1.62	0.10	1.63
19	0.88	8.98	2.93	76.4	15.0	146	17.0	16.3	0.26	1.47	0.13	1.30
20	1.21	8.86	1.04	77.1	17.1	134	14.1	15.1	0.24	1.05	0.07	0.87

**Mean**	**0.88**	**7.43**	**2.66**	**62.6**	**13.5**	**131**	**15.2**	**17.9**	**0.22**	**1.04**	**0.09**	**1.07**
***SD***	**0.31**	**1.46**	**1.18**	**12.6**	**5.41**	**20**	**2.4**	**2.0**	**0.07**	**0.20**	**0.04**	**0.20**

*^1^LCAT concentration was measured using an ELISA kit provided by Daiichi Pure Chemicals, Japan, according to the procedures described by Kobori et al. (2002). ^2^For measurement of ECER, paired samples of plasma and lymph were incubated *in vitro* either in crushed ice or at 37°C for 3 h, and then rapidly cooled to 0–4°C in crushed ice. The rate of esterification of cholesterol was calculated from the decrease in the concentration of UC (Albers et al., 1986) measured enzymatically (Nanjee and Miller, 1996). ^3^LCAT activity was measured using an optimal proteoliposome substrate of apo AI, PC and ^14^[C]-labeled UC (molar ratio 1:300:10; Albers et al., 1986). ^4^In each sample of lymph or plasma, LCAT specific activity was calculated by dividing LCAT activity by LCAT concentration. ^5^Apo AI concentration was measured by immunoelectrophoresis as described by Nanjee et al. (2000a). ^6^The concentration of UC was quantified enzymatically (Nanjee and Miller, 1996). LCAT, lecithin:cholesterol acyltransferase; ECER, endogenous cholesterol esterification rate; apo AI, apolipoprotein AI; sp activity, specific activity; UC, unesterified cholesterol; SD, standard deviation. The values underlined are the highest and lowest in each column.*

### LCAT Activity

**FIGURE 1:**
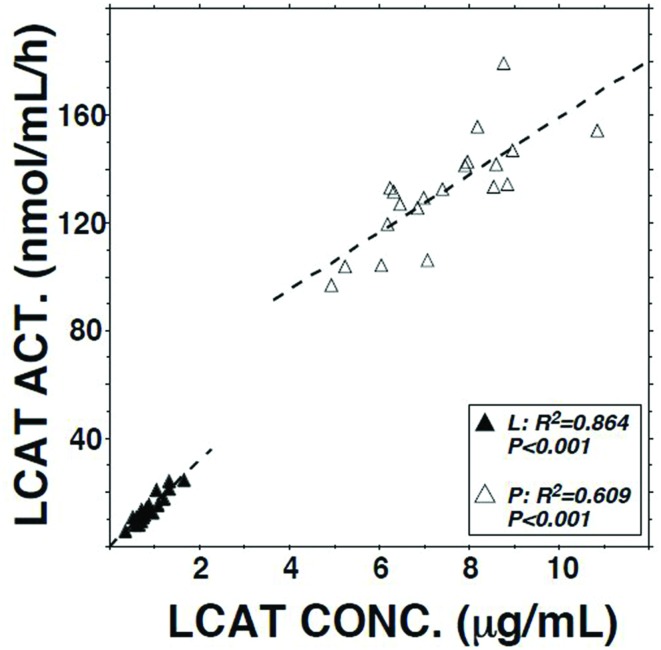
**Relation of LCAT activity to LCAT concentration in plasma and lymph samples from 20 subjects.** Assays were performed as described in **Table 1**.

LCAT activity in lymph assayed using a radio-labeled proteoliposome substrate averaged 10.3% (*P* < 0.01) of that in plasma (**Table 1**). It was positively correlated with both lymph LCAT concentration (*r* = +0.93, *P* < 0.001; **Figure 1**) and lymph apo AI concentration (*r* = +0.83, *P* < 0.001), but not with plasma LCAT activity (*r* = +0.12). The correlation coefficient between plasma LCAT activity and plasma LCAT concentration was +0.78 (*P* < 0.001; **Figure 1**).

### LCAT Concentration

Lymph LCAT concentration averaged 11.8% (*P* < 0.01) of plasma LCAT concentration (**Table 1**). It was not significantly correlated with plasma LCAT concentration (*r* = +0.26, *P* = 0.27), but was correlated with lymph apo AI concentration (*r* = +0.80, *P* < 0.001).

### Specific Activity of LCAT

The specific activities of LCAT in lymph and plasma were calculated by dividing the result obtained for LCAT activity by that for LCAT concentration in the same sample. On average, the specific activity in lymph was slightly lower than that in plasma (*P* < 0.001; **Table 1**).

### Apo AI and UC Concentrations

In agreement with previous studies, lymph apo AI concentration averaged about 21% of plasma apo AI concentration (*P* < 0.001; **Table 1**). The two were positively correlated (*r* = +0.54, *P* = 0.013). The concentration of UC in lymph averaged 8.4% (*P* < 0.001) of that in plasma (**Table 1**).

### Effect on ECER of Mixing Lymph with Native or Heat-Inactivated Plasma

**FIGURE 2:**
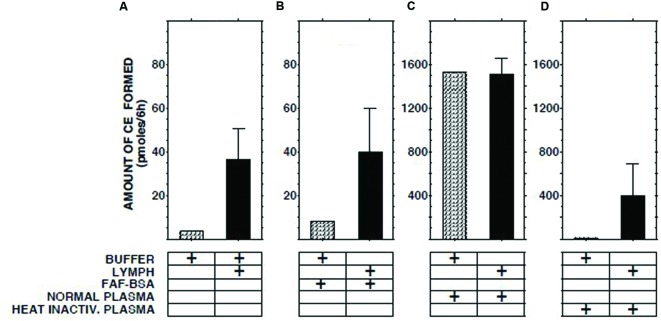
**Effect on ECER of mixing lymph (100 μl) from five subjects with an equal volume of pooled normal plasma, heat-inactivated pooled plasma, or fatty acid-free albumin (final conc 50 mg/ml).** Note that the scale of the vertical axes in panels **(A,B)** differs from that in panels **(C,D)**. The buffer used was 50 mmol/l Tris-buffered saline, pH 7.4. Statistical differences are described in the text. CE, cholesteryl esters. Results are means and SEM.

The ECER observed with various mixtures of lymph, buffer, buffer containing fatty acid-free albumin, native human plasma, and heat-inactivated human plasma are presented in **Figure 2**. The result obtained with a mixture of lymph and native plasma was essentially identical to that obtained with a mixture of similar volumes of buffer and native plasma, providing evidence that lymph does not contain a soluble inhibitor of cholesterol esterification. The ECER observed during incubation of lymph with heat-inactivated human plasma (i.e., in which LCAT had been inactivated by incubation for 30 min at 56°C, ref 20) was 11-fold greater than that observed with a mixture of similar volumes of lymph and buffer, demonstrating that the enzyme in lymph was functional and able to catalyze the esterification of UC in plasma HDLs. In the same series of incubations, addition of fatty acid-free albumin had no effect on the ECER in lymph (**Figure 2**).

### Effect of Adding Recombinant Human LCAT

**FIGURE 3:**
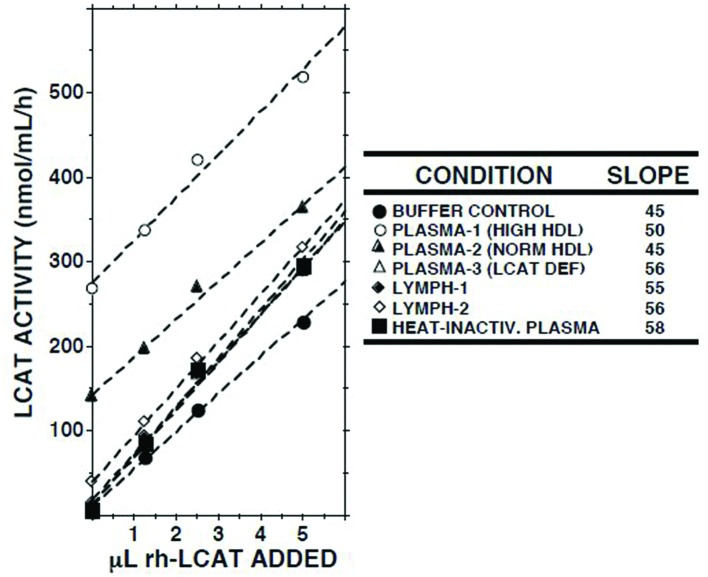
**Effects of adding recombinant human LCAT (rh-LCAT; 12.5 μl) on LCAT activity in samples of plasma and lymph from subjects with normal and high HDL cholesterol concentrations, plasma from a subject with familial LCAT deficiency (Nanjee et al., 2003), plasma in which LCAT had been heat-inactivated (30 min at 56°C), and Tris-buffered saline, pH 7.4.** LCAT activity was measured as described in the footnote to **Table 1**. Human rh-LCAT was a generous gift from Dr John Parks (Miller et al., 1996).

When increasing amounts of recombinant human LCAT were added to lymph, a linear increase in LCAT activity was recorded (**Figure 3**). The slopes of the regression lines were similar to those observed when LCAT was added to buffer, normal plasma, plasma from a subject with familial LCAT deficiency (Nanjee et al., 2003), or heat-inactivated plasma.

### Effect on ECER of Triglyceride-Rich Lipoproteins

**FIGURE 4:**
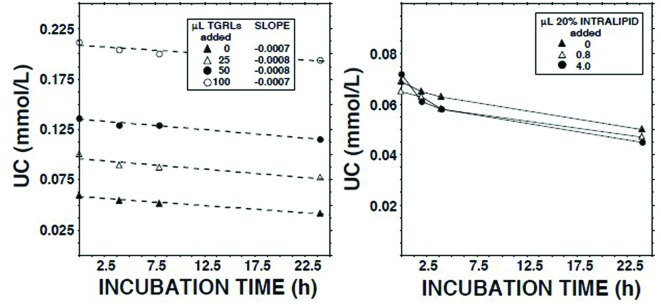
**Effects on the unesterified cholesterol concentration (Nanjee and Miller, 1996) in lymph during 22.5 h of adding Intralipid 20% (Fresenius Kabi, Clayton, NC; containing 200 g soybean oil/l; right panel) or the *d* < 1.006 g/ml fraction of fasted normal human plasma (plasma triglycerides, 1.4 mmol/l)**.

We have reported that human lymph contains both CE transfer protein (CETP) and CETP activity (Miller et al., 2013). The principal recipient particles for cholesteryl esters (CEs) transferred from HDLs by CETP in plasma are triglyceride-rich lipoproteins (TGRLs) and low density lipoproteins (LDLs; Fielding and Fielding, 1981). As LCAT activity is inhibited by CEs (Chajek et al., 1980), and lymph contains almost no TGRLs and few LDLs (Nanjee et al., 2000b, 2001), we tested the hypothesis that LCAT in lymph is inhibited as a consequence of retention of CEs in spheroidal HDLs. However, addition of neither the *d* < 1.006 g/ml fraction of normal human plasma nor Intralipid 20% increased the ECER (**Figure 4**).

### Effects of Adding Lipid-Free Apo AI, Apo AI/PC disks, or Apo AI/PC/UC disks

**FIGURE 5:**
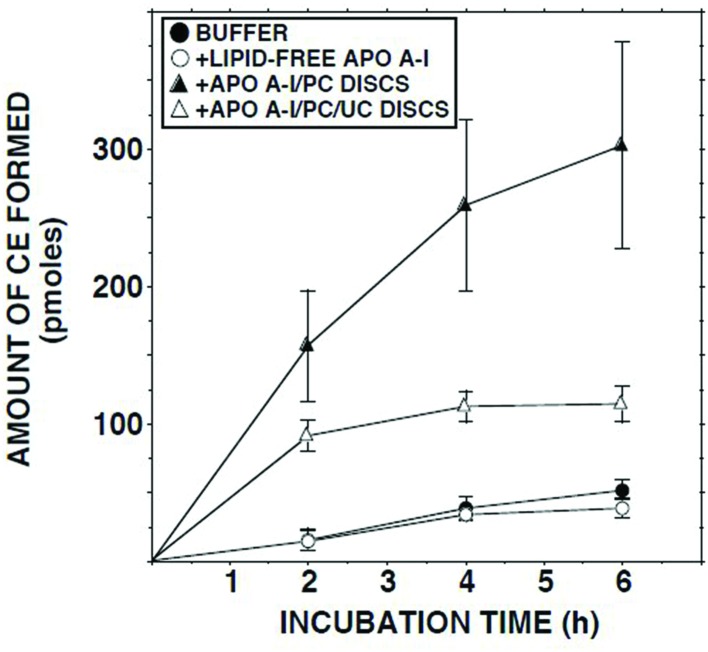
**ECER in lymph samples from five subjects as a function of time following addition of Tris-buffered saline, fatty acid-free albumin (final conc 50 mg/ml), apo AI/PC disks (1:150 molar ratio; final conc 0.66 mg apo AI/ml), or apo AI/PC/UC disks (1:150:10, final conc 0.66 mg apo AI/ml)**. Each incubation mixture was composed of 100 μl lymph or Tris-buffered saline plus 100 μl of buffer or the test material. At the end of the incubation, 10 μl aliquots were analyzed in duplicate for UC mass (Nanjee and Miller, 1996). The disks were provided by Central Laboratory, Swiss Red Cross, Bern, Switzerland. Results are means and SEM.

Addition of delipidated human apo AI to lymph had no effect on the ECER (**Figure 5**). By contrast, addition of discoidal proteoliposomes composed of human apo AI in association with PC increased the ECER during the first 2 h of incubation by 10-fold (**Figure 5**). When similar incubations were carried out using disks containing UC in addition to apo AI and PC, the ECER was increased by only 6-fold (**Figure 5**).

## Discussion

This is the first study of the mechanism underlying the very low rate of cholesterol esterification in human interstitial fluid relative to plasma. We found that afferent lymph, which is representative of interstitial fluid (Michel and Curry, 1999; Nanjee et al., 2000b, 2001), contains active LCAT enzyme. As expected, LCAT concentration was much lower than in plasma. Nevertheless, when lymph was added to heat-inactivated plasma, the enzyme catalyzed the esterification of UC at a rate of more than 10-fold that in lymph alone, showing that LCAT concentration was not the rate-limiting factor.

No evidence was obtained for a soluble inhibitor of LCAT in lymph. First, when lymph was added to plasma, the ECER was not reduced relative to that observed in plasma alone. Second, when increasing concentrations of recombinant human LCAT were added to lymph, the increments in enzyme activity were similar to those observed when the same masses of LCAT were added to normal, heat-inactivated, or LCAT-deficient human plasma.

The much lower concentration of UC in lymph than in plasma raised the question of whether it was limiting the ECER. However, this hypothesis was not compatible with the fact that the lymph/plasma ratio in ECER was much lower than that in UC concentration.

Thus, our evidence suggested that the low esterification rate in lymph HDLs is owing to a property that renders them a poor substrate for LCAT. This hypothesis was supported by the finding that the initial ECER in lymph was increased 10-fold when apo AI/PC disks were added. By contrast, we have previously reported that addition of similar disks to plasma had no effect on ECER (Nanjee et al., 1999). We cannot determine from our data whether the additional esterification in lymph occurred in the disks or the lymph HDLs. LCAT exists in association with HDLs, and the affinity of discoidal HDLs for the enzyme exceeds that of spheroidal CE-rich HDLs (Kosek et al., 1999). The catalytic efficiency of LCAT is also normally greater on discoidal HDLs (Kosek et al., 1999). Thus, it is likely that some UC and LCAT moved from the lymph HDLs to the disks, followed by esterification of UC on the disks. A similar sequence may explain the esterification observed when lymph was mixed with heat-inactivated plasma, the lymph HDLs acting as a donor of active LCAT to drive the reaction in plasma HDLs. An alternative possibility is that the disks (or plasma HDLs in the case of heat-inactivated plasma) have a greater affinity for a dissociable inhibitor of LCAT than lymph HDLs, thereby ‘cleansing’ the latter and enabling the reaction to proceed. Theoretical possibilities for inhibitors include oxidized lipids (Bielicki et al., 1996; Kamiyama et al., 1998) and lysophosphatidylcholine (Smith and Kuksis, 1980). However, the failure of delipidated apo AI and fatty acid-free albumin, which bind oxidized lipids and lysophosphatidylcholine (Navab et al., 2000; Ek-Von Mentzer et al., 2001; Matsumoto et al., 2007), to produce even a small increase in ECER argues against these possibilities.

As LCAT is inhibited by CEs (Fielding and Fielding, 1981), the hypothesis was tested that the ECER in lymph is suppressed by retention of CEs in HDLs secondary to the very low concentrations of apo B-containing lipoproteins. While this seemed unlikely given that lymph HDLs have a greater UC/CE ratio and lower CE/apo AI ratio than plasma HDLs (Nanjee et al., 2000b, 2001), the possibility remained that it was a contributing factor in some HDL subclasses. However, the failure of the *d* < 1.006 fraction to increase the ECER despite the presence of CETP in lymph (Miller et al., 2013), albeit in low concentration, argued against this mechanism.

The possibility that esterification in lymph HDLs is suppressed by sphingomyelin, which inhibits LCAT (Bolin and Jonas, 1996), also merits consideration, as the sphingomyelin/PC molar ratio is greater in lymph than in plasma (Nanjee et al., 2000b). However, the difference in ratio (0.33 vs. 0.28; Nanjee et al., 2000b) appears too small to explain such a large difference in ECER.

On the basis of studies of apo AI epitopes in suction blister fluid, Wong et al. (1992) concluded that apo AI in interstitial fluid does not function as a cofactor for LCAT owing to a change in conformation. This explanation would be compatible with some of our findings. For it to be correct, however, the same change in conformation would need to involve all HDL subclasses in lymph: CE-rich alpha HDLs, lipid-poor pre-beta HDLs, and discoidal HDLs (Nanjee et al., 2000a, 2001; Miller et al., 2013). This would be surprising given that the conformation of apo AI already differs among them. This mechanism would also not explain why apos E and AIV, both of which are present in lymph (Nanjee et al., 2000b) and have cofactor activity for LCAT (Bisgaier et al., 1987; Gong et al., 1989; Emmanuel et al., 1994), do not maintain esterification. The possibility exists that the findings of Wong et al. (1992) were due to tissue disruption and release of proteases during production of the blisters.

In contrast to the apo AI/PC disks, we found that disks with the same apo AI/PC ratio that also contained UC were much less effective in raising ECER. This would be consistent with suppression of cholesterol esterification in lymph HDLs being due to substrate inhibition. Substrate inhibition of LCAT at high UC/PC ratios in HDLs, achieved by exposure to phospholipase A2 (Chollet et al., 1986) or enrichment with UC (Simard et al., 1989), has been observed *in vitro*. In studies with sonicated dispersions of UC and PC, Nichols and Gong (1971) also found that net esterification and initial reaction rate decreased with increasing UC/PC ratio.

Thus, our findings suggest that esterification of UC in interstitial fluid HDLs is suppressed as a consequence of substrate inhibition by UC transferred from cells via ABCA1 transporters. When lymph HDLs enter blood via the lymphatic vessels, the UC redistributes among plasma lipoproteins, thereby reducing the inhibition and enabling esterification to proceed.

It is interesting to speculate on the function of substrate inhibition in interstitial fluid. Kinetic analyses of apo AI after intravenous apo AI/PC disks have indicated that HDLs remain in the extracellular matrix on average for about 29 h before entering the lymph vessels (Hovorka et al., 2006). Lymph is virtually devoid of TGRLs (Nanjee et al., 2000b, 2001), the principal recipients in plasma of CEs transferred via CETP from HDLs. Thus, if UC delivered to HDLs from cells via ABCA1 transporters were continuously esterified, the increase in the size of the particles consequent upon retention of CEs might impede their passage through the interstices of the matrix. An alternative or additional function might be to limit the production of lysophosphatidylcholine in the presence of albumin concentrations that are only one third of those in plasma (Nanjee et al., 2000b), thereby protecting tissues from its detergent and pro-inflammatory activities (Matsumoto et al., 2007).

Our experiments used lymph collected from a vessel that drains skin, connective tissue, and adipose tissue. As other investigators have shown that reverse cholesterol transport from arterial wall macrophages also occurs via lymphatic vessels (Lim et al., 2013; Martel et al., 2013), our findings have implications for strategies to prevent atherosclerosis by modulation of HDL metabolism. If LCAT is inhibited upon entering interstitial fluid in the artery wall, raising its concentration in plasma pharmacologically or by intravenous infusion might not increase eﬄux of UC from arterial macrophages, its effect being limited to cells exposed directly to plasma (e.g., erythrocytes, liver macrophages, spleen cells). This might explain why LCAT transgenesis has failed to protect mice from atherosclerosis (Bérard et al., 1997; Föger et al., 1999). Brousseau et al. (2000) found that LCAT transgenesis also had no effect on atherosclerosis in LDL receptor negative rabbits. Although it did reduce lesions in cholesterol-fed LDL receptor positive rabbits, this was dependent on an associated reduction of LDL concentration. Van Craeyveld et al. (2009) observed no significant effect of hepatocyte-directed adenoviral rabbit LCAT gene transfer on atherosclerosis in cholesterol-fed rabbits.

## Author Contributions

NM conceived and organized the study and was the PI, contributed to the interpretation of the results, and wrote the first draft of the manuscript. WO performed the lymph vessel cannulations, and contributed to the writing of the manuscript. IM undertook the statistical analysis of the data, contributed to their interpretation, and contributed to the preparation of the manuscript. MN designed the laboratory experiments, performed all the experiments and assays, and contributed to the writing of the manuscript.

## Conflict of Interest Statement

The authors declare that the research was conducted in the absence of any commercial or financial relationships that could be construed as a potential conflict of interest.
